# Key tips to shift student perspectives through transformative learning in medical education

**DOI:** 10.1186/s12909-025-06754-2

**Published:** 2025-02-07

**Authors:** Efraim J. Hart, Merel H. de Heer-Koster, Maria van der Harst, Joyce L. Browne, Fedde Scheele

**Affiliations:** 1 Department of medical education, OLVG Hospital, Amsterdam, Netherlands; 2 Athena Institute, Faculty of Science, VU, Amsterdam, Netherlands; 3 Amsterdam UMC, Location Vrije Universiteit Amsterdam, Research in Education, Amsterdam, Netherlands; 4 Centre for Global Challenges, Faculty of law, economics, governance & organization, Utrecht University, Utrecht, Netherlands; 5 Julius center for health science and primary care, UMC Utrecht, Global Public Health & Bioethics, Utrecht, Netherlands; 6 ACTA, Academic Center for Dentistry University of Amsterdam, Amsterdam, Netherlands

**Keywords:** Transformative learning, Change agents, Practical tips, Pedagogical innovation, Curriculum reform

## Abstract

Healthcare systems are increasingly complex, facing challenges such as rising costs, aging populations, and persistent health inequities. These challenges demand that medical education adapts, to equip health professionals with the competencies required to navigate and transform complex systems. This is where transformative learning theory can play a crucial role. This article offers twelve practical tips to integrate transformative learning in medical education, emphasizing the need for a shift from traditional educational approaches to more transformative methods. These tips focus on educating learners on the principles of transformative learning, encouraging critical reflection, fostering interdisciplinary collaboration, addressing the hidden curriculum, and creating a supportive learning environment. By implementing these tips, medical education can better prepare healthcare professionals to act as change agents, contributing to the long-term sustainability and effectiveness of healthcare systems. The potential impact of these tips on medical education is substantial, as they provide a pathway toward a more responsive, ethical, and sustainable future of healthcare. Clinical trial number: Not applicable.

## Background

Healthcare is in transition and faces major challenges. These include rising costs, aging populations, workforce shortages, a large ecological footprint, and unevenly distributed access to care resulting in persisting health inequities [1, 2]. Healthcare systems worldwide are struggling to keep up with all the changes as healthcare systems become increasingly complex, and more demands are placed on healthcare workers [3]. There is growing consensus among international government agencies, scientific, and non-governmental institutions that the world is rapidly changing, and health systems need to transform to keep pace with these changes and promote long-term well-being and environmentally sustainable development [4, 5]. This imposes an important new requirement on how we train health professionals, namely; to have more immediate impact through enhanced effectiveness and direct action in addressing complex problems and promote transformation in society [3–6].

Yet, in the words of Frenk et al., *“education in healthcare has not kept pace with these systemic challenges”* [3]. Competencies related to navigating complexity, systems thinking and adequate action are almost entirely lacking in medical educational programs [3, 5, 7], resulting a lack of crucial competencies to address current systemic challenges upon graduation [3, 5, 8]. Increasingly, educators argue that the mission within education must be redefined, and the way students are taught must be adapted to enable students to tackle complex challenges, take action, and contribute to the societal transitions taking place [3–5].

A fundamental, transformative shift in thinking, values, and actions in medical education is therefore necessary to prepare health professionals to take up change agent roles in the rapidly changing healthcare systems [3, 4, 9, 10]. The realization of this vision requires a series of educational and institutional reforms, with transformative learning as the proposed process and outcome [3]. Transformative learning focuses on deep fundamental shifts in an individual’s worldview [11], leading to new ways of being in the world [12]. This pedagogical approach goes beyond traditional methods, focusing on deep, meaningful learning experiences that change how students think and practice. Focus on this level seems to be absent in medical curricula and at most training sites too often [9, 13–16], or it is occurring, but goes unrecognized in some settings [10] while the need for transformative learning in medical education has increased [3]. As Mezirow states, *“if circumstances permit*,* students participating in a transformative learning process move towards a more inclusive*,* self-reflective*,* and integrative frame of reference”* [17, 18]. Theorists agree that transformative learning begins when individuals critically reflect on their assumptions and on what they consider to be true or right [19]. Ultimately, transformative learning in medical education supports the training of competent, compassionate, and adaptive healthcare professionals [17–19] and can help bridge the gap between the current state of medical education and the needs of healthcare systems, enabling professionals to become change agents in their fields.

## Methodology

The tips presented in this article were developed through a multi-faceted approach. This process included:

- **Literature Review**: A comprehensive review of transformative learning theory and its applications in medical education informed the development of these tips.
- **Course Design**: Insights were drawn from designing transformative learning courses for students and health professionals.
- **Course implementation and improvement**: During this phase, we encountered various challenges and often referred back to the literature to reflect on these barriers and experiences, as well as participant reflections. Several challenges were met and addressed using insights from global literature and collaborative discussions. The literature was continuously evaluated and updated based on new experiences during course implementation.
- **Participant Reflections**: Anecdotal feedback and reflections from learners provided valuable input, helping identify common challenges and effective strategies.

Research indicates that educating physicians to provide high-value, cost-conscious care is most effective when it integrates three key components; knowledge transmission, reflective practice and a supportive environment. “*These factors should be considered when educational interventions are being developed”* [20]. Accordingly, we used these three factors as themes in our classification of the transformative learning tips.

### Theme 1: knowledge transmission

This theme focuses on the importance of educating learners about the foundations and significance of transformative learning in medical education. It also focuses on helping students understand how transformative learning differs from other levels of learning and why it is crucial for professional growth and improving healthcare systems. It is based on transformative learning theory and insights from courses designed to integrate these concepts.

#### Educators are encouraged to incorporate an early session on transformative learning principles and their importance

Understanding that transformative learning is fundamentally different from other forms of learning is crucial for transformative learning to take place (Table 1) [3]. It is especially important in a rapidly evolving world, where the ability to reassess and transform one’s understanding is essential for addressing complex challenges and driving innovation [3, 21]. Additionally, it should demonstrate how transformative learning contributes to enhanced patient care and a positive organizational culture. By understanding the value of transformative learning, students will be better equipped to engage with the process, recognizing its relevance to their professional development and its broader impact on the healthcare system [17]. Incorporate an introductory session or workshop early in the curriculum to educate students on the principles of transformative learning and its importance for both individuals and healthcare organizations. This session should explain the theory behind transformative learning, highlighting its role in fostering critical thinking, personal growth, and adapting to new challenges in healthcare and other environments.

**Table 1:** A merge of the levels of learning according to Frenk et al. [3] and Sterling [6]

Levels of learning [3, 6]	Objectives	Response	Outcome
1. Informative (bolt-on)	Information, skills	Doing things better	Experts
2. Formative (build-in)	Socialization, values	Doing better things	Professionals
3. Transformative (transformation)	Leadership attributes	Seeing things differently	Change agents

**1**: informative, ‘bolt-on’ through this response, the dominant paradigm maintains its stability. **2**: formative, ‘build-in’ of ideas to the existing system, through which the system itself experiences significant change. This is critically reflective, adaptive response, or second-order change, where paradigmatic assumptions are called into question. **3**: transformative: this is a deep, conscious reordering of assumptions which leads to paradigm change

#### Assist students in finding a disorienting and personal dilemma

In transformative learning, learners have to be challenged to question previous habits, beliefs, and assumptions [22, 23]. Critical reflection is usually triggered when a disagreement occurs between an individual’s current assumptions and a newly presented point of view. Mezirow referred to this disagreement as a ‘disorienting dilemma’ [17, 24], the initial phase of transformative learning. For transformative learning it is important for learners to know if they are already experiencing a disorienting dilemma or if attention is needed for this dilemma for the transformative process to take place. Students should engage in critical reflection on this experience, examining their emotions, underlying assumptions, and the broader implications for their future practice. This personalized approach promotes deep self-reflection and critical thinking, making the learning experience more relevant and transformative for the student [23]. Encourage students to identify and delve into a personal dilemma they have faced during their clinical training or education. This might involve a challenging interaction with a patient, a complex ethical decision, or a situation that elicited a strong emotional response or uncertainty.

#### Focus on societal responsiveness, emphasizing ethical and cultural competence

To help learners to be reflective and find their disorienting dilemma, it is important to incorporate training on (bio)ethics and cultural competence, so students learn to question social arrangements, structures and their own views [25, 26]. Encouraging learners to frequently question the healthcare system they are part of and discussing ethical dilemmas and cultural sensitivity helps students develop empathy and moral reasoning [3, 13]. Learners preparing to enter the work floor or working as a health professional should be exposed to ethics, social sciences and concepts of social justice. This exposure is essential for them to perform effectively and responsible within the healthcare systems [3]. This will help students to appreciate that while the systems in place may appear naturally, these are in fact produced and subject to change [6]. As Sterling et al. explains, “*the logic of this is that learning within paradigm does not change the paradigm*,* whereas learning that facilitates a fundamental recognition of paradigm and enables paradigmatic reconstruction is by definition transformative*” [6]. A practical example of an approach to fostering societal responsiveness is having a program director regularly discuss current events, newspapers, and health politics with the learners [13].

### Theme 2 reflective practice

This theme focuses on the importance of experiential learning, fostering interprofessional collaboration, and integrating reflective practices. It argues that learning should go beyond information delivery, engaging students on a personal and emotional level to drive deeper understanding and perspective change. The recommendations come from literature and practical experiences in implementing such activities in medical education.

#### Provide suitable experiences for experiential learning

Learning becomes most effective when learners actively participate, take initiative, and engage with real-life experiences that challenge them cognitively, but also engage them personally and emotionally. The learner must make sense of the events experienced. The combination of actively experiencing something, especially if it is accompanied by intense emotions, may result in long lasting learning. This type of learning is best described as experiential learning; which can consist of learning by doing, thinking about, and integration of lessons learned into everyday behaviors [27, 28]. In summary, to make learning impactful, focus on providing experiences where learners can actively participate, reflect, and engage emotionally. This approach helps create meaningful, lasting lessons that learners can integrate into their daily lives.

#### Foster collaboration and perspective sharing across disciplines

The ability to understand competing demands and work with the needs, interests, and perspectives of others is crucial in reconciling tensions and dilemmas [17, 21, 24]. In today’s interconnected world, addressing challenges involves managing trade-offs, such as those between the autonomy of the patient and the expertise of the health professional [29] and the immediate financial gains of a business against the long-term environmental and social impacts of its operations. It is important that learners recognize that complex problems, like in sustainable development, may have multiple solutions. By holding opposing ideas in balance, learners can deepen their understanding of different perspectives and strengthen their arguments in conflicts and dilemmas [21].

Exposure to diverse viewpoints allows students to grasp the complexity of various issues and develop a more nuanced understanding of challenges in their field [3, 13]. Designing activities that challenge students to think critically and develop strategies for change is crucial. Moving away from passive lectures and engaging students through active learning strategies such as group discussions, case studies, and interactive workshops can foster deeper understanding and encourage perspective sharing across disciplines [24, 30].

A key example of perspective sharing in action is interprofessional learning, where students from different healthcare backgrounds and disciplines collaborate in cases to expand their understanding, question their assumptions, and develop more holistic approaches to patient care [3, 7]. Exposure to varied professional viewpoints helps students question their assumptions and broaden their perspectives [3].

#### Assign a jester

Implement role-playing exercises in which one of the learners is designated as the provocateur or “Jester” within the group [30, 31]. This role of the Jester is to question assumptions, challenge prevailing ideas, and introduce alternative perspectives during the discussion [31, 32] and express dissent without fear of retribution [31]. The ability to dissent without direct consequences makes them invaluable, especially in situations where conformity is the norm [33, 34]. By having someone in a group playing this role, a group of learners can reflect on and learn to critically assess their own viewpoints and those of others, which is essential for developing a deeper understanding of complex medical issues [35]. This method not only enhances critical thinking but also prepares students to think about ethical issues [35] and navigate diverse perspectives in real-world healthcare settings. It is crucial for learners to practice recognizing different frames of reference and to apply their creativity in reframing problems from new perspectives [17, 24].

#### Integrate reflective practice into the curriculum

Encourage learners to question and critically reflect on their experiences, clinical decisions, and interactions with patients. Critical reflection helps them understand their biases, assumptions, and areas for improvement [17, 24, 36, 37]. Mezirow elaborates that “*transformations in frames of reference take place through critical reflection and transformation of a habit of mind*,* or they may result from an accretion of transformations in points of view*” [17].

This can be facilitated through reflective writing assignments or group discussions where learners are encouraged to share their views openly, without immediate judgment [30]. By first recognizing and understanding these perspectives, educators can more effectively support students in critically evaluating and potentially transforming their underlying habits of mind, fostering deeper and more meaningful learning experiences [17]. Educators can identify and model how professional values are reflected in reflective writing. For instance, this involves guiding learners to align their reflective writing with disciplinary values, such as safety, effective communication, and professionalism, or other values outlined in their core competency frameworks [38].

**Fig. 1:**
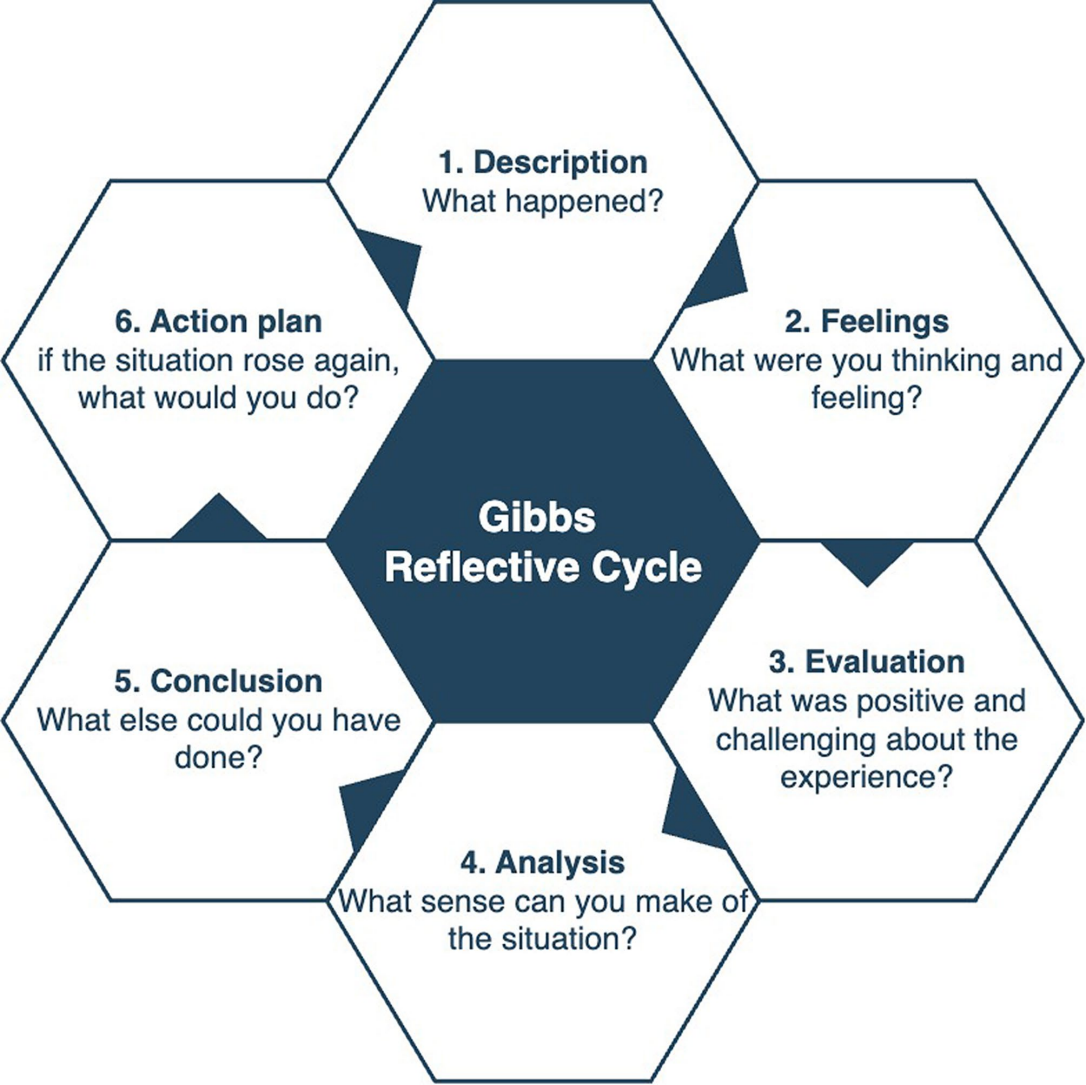
Based on Gibbs’ Reflective Cycle [28, 39]

A well-known example of a reflective cycle was developed by Gibbs’ and it consists of six stages (Fig. 1) [28, 39]. There are several suitable variations of reflective cycles published [37]. These reflective cycles can help learners to evaluate their experiences in relation to professional standards and disciplinary values [38].

#### Train students to participate in deeper discourse

Learners engage in discourse to confirm and clarify what is being communicated. Discourse is important for validating both what learners understand and how they understand it, or for reaching a well-informed judgment about a belief. In this way, learning becomes a social process, with discourse playing a central role in creating meaning [17]. Implement training sessions that teach learners how to engage in deeper discourse, encouraging them to delve into underlying assumptions, values, and broader implications in medical practice [17]. This can be achieved through dialogue beyond engaging in superficial conversation [17, 30]. Educators can create opportunities for learners to practice discourse by incorporating techniques such as structured debates, or facilitated small-group discussions where students practice expressing their thoughts and critically evaluating the perspectives of others [23] and to evaluate meanings that align with core disciplinary values [40]. There are various techniques to evaluate these meanings, which can be very helpful for reflection in transformative learning [40].

### Theme 3: a supportive environment

This theme focuses on creating an environment that supports transformative learning. It stresses the need to uncover and address the “hidden curriculum,” promote open dialogue and reflection, and continuously assess and adapt the curriculum to remain aligned with evolving societal and healthcare needs. This section builds on research on education and organizational culture.

#### Focus on a deep understanding of patient needs and narratives

Medical textbooks often do not give a deep enough understanding of the needs and underlying issues of the specific population learners are working with [41]. Also, medical education often emphasizes emotional detachment, clinical neutrality, and a strong focus on the science of medicine [41, 42]. This can lead students to mistakenly believe that avoiding interpersonal engagement with patients is endorsed, which in turn contributes to a decline in empathy among medical students, residents and physicians [41, 42]. As empathy declines during medical school, it is important to include patient narratives and storytelling in the curriculum to illuminate the human behind the condition, encourage empathy and a deeper understanding of patient experiences [42]. Role models must repeatedly explain that overemphasis on evidence can result in “*culturally and personally suboptimal care for the individual patient*”, also because of epistemic injustices and systemic biases in our production of knowledge [43]. Moreover, they must show that effective decision-making involves a multi-perspective balancing between the patients’ views, evidence from the literature, clinical experience, and understanding of organizational influences. These firmly rooted approaches of health care are fundaments for a transformative learning culture [41].

A practical way to achieve this deeper understanding is through problem-posing learning. In clinical case study sessions, rather than merely presenting the case and expecting learners to arrive at a diagnosis, encourage them to identify and discuss the underlying issues, ethical dilemmas, and social factors that may influence patient care. Facilitate group discussions where students pose their own questions about the case, explore multiple perspectives, and collaboratively seek solutions [44, 45]. The clinical work floor management has to ensure time for such meetings concerning real patients.

#### Unhide and address the hidden curriculum

The key factors shaping the identity of health professionals often stem from the hidden curriculum, which operates outside the formal curriculum and is less overtly recognized [46]. While there have been efforts to implement transformative learning all over the medical field and a focus on more holistic medical education, training in leadership, advocacy, change agency and different roles for health professionals [3, 47], this is often not the focus in the hidden curriculum. The hidden curriculum often focusses on acquiring and assimilating a large knowledge base (informative learning, Table 1), with a big focus on the medical expert role [16]. Academic organizations acknowledge that the hidden curriculum plays a crucial role in either promoting or hindering the roles of advocate and professional [48], roles that are essential for transformative learning [17, 24, 49]. Despite efforts to promote patient-centered care [41] and empathy [42], trainees are frequently exposed to conflicting messages from the hidden curriculum [41]. The hidden curriculum often reinforces hierarchies and inequalities within healthcare, giving certain groups advantages [41, 48, 50]. Addressing and making the hidden curriculum visible is a critical but challenging task. To effectively address it, institutions must recognize the influence of the hidden curriculum and work to resist its negative impacts [41, 48]. A deeper understanding of its specific elements, and their relationship with professionalism, is needed to ensure the delivery of compassionate, patient-centered care. All healthcare professionals must be aware of its effects [41, 48, 50]. Once more, both educational staff and clinical work floor management have to arrange regularly structured meetings to reflect on the hidden curriculum and action to be taken to avoid wrong messages coming from it. These efforts add to an optimal culture of transformative learning.

#### Evaluate and adapt curriculum regularly

A culture of transformative learning requires reflectivity to be fully integrated through a shared vision, where the need for change is recognized as a continuing process [51]. Regularly assess and update the curriculum to ensure it meets the evolving needs of medical education and of society. Gather feedback from students and faculty to make informed improvements and stay relevant with current medical practices and societal changes [3]. When assessing the curriculum, recognize that competency-based medical education (CBME) will often need to be supplemented to address transformative learning, because of usually rigidly defined or interpreted learning outcomes like Entrustable Professional Activities (EPAs) [52]. In CBME opportunities for transformative learning are provided by partially open training plans [52], but with the hidden curriculum in full effect [41, 48, 50], it is important to not only provide open training plans for more room for transformative learning, but to make transformative learning an explicitly important part of the curriculum [9].

#### Create an open culture as a fundament for transformative learning

A supportive and non-threatening environment encourages learners to take risks, ask questions, and reflect on their experiences [53, 54]. Facilitate open discussions and ensure that feedback is constructive and respectful. There is an emancipatory potential, but that also carries a potential of alienation [55]. To create a safe space, supervisors and learners need to withhold judgment and work to establish mutual respect, trust, and a comfortable rapport with each other [24, 53, 54]. Also, it involves a culture of continuous learning and open communication, where supervisors serve as supportive role models, maintaining high ethical standards, and refraining from power abuse, and blame [54]. Efforts should be made to empower learners as agents of change, providing them with the status, authority, and skills necessary to contribute to positive societal transformation. While not every learner needs to become a social reformer, there should be no artificial barriers preventing professionals from exercising their social agency if they choose to do so. The choice to engage in social change is a personal one, but in a transformative learning culture, the opportunity should be accessible to all [3].

Recognizing cultural differences, such as those highlighted in Hofstede’s and Meyer’s frameworks, is equally important for the successful implementation of transformative learning [56–58]. Hofstede’s dimensions help us understand how cultural variations influence group dynamics and open culture [56, 57]. In cultures with high power distance, *“the extent to which the less powerful members of institutions expect and accept that power is distributed unequally”* [56], hierarchical structures may discourage open dialogue, affecting the potential for collaborative learning and critical thinking [53]. It is important to acknowledge the cultural differences regarding power structures and to address the influence of the existing hierarchy and the conditions for an open culture [54] in relation to transformative learning.

## Conclusion

Implementing transformative learning in a curriculum is feasible and the necessity for transformative learning has increased due to the rapidly evolving challenges within healthcare systems and societies. Many current curricula do not adequately integrate key competencies needed for transformative learning. To equip healthcare professionals as effective change agents, medical education must move beyond traditional, informative and formative learning towards a transformative approach that encourages deep reflection, interprofessional collaboration, and a strong sense of societal responsibility. To achieve this we focused on knowledge, application and cultivation of transformative learning. Achieving a transformative learning approach requires continuous curriculum evaluation, the cultivation of an open learning environment, and a critical examination of the hidden curriculum. The tips presented may lead to implementation of transformative learning in medical curricula. It remains important to consider cultural differences within the context of different countries and fields when implementing the tips.

Future research should include the development and evaluation of empirical frameworks to measure the impact of transformative learning interventions on competencies, behavioral change and system transformations. As transformative learning has the potential to redefine medical education and contribute to more compassionate, ethical, and effective healthcare systems, longitudinal studies could explore how these pedagogical changes can have a sustained influence on learners’ competencies, professional behavior, and societal impact.

## Data Availability

No datasets were generated or analysed during the current study.
